# Impact of PCSK9 Inhibition on Proinflammatory Cytokines and Matrix Metalloproteinases Release in Patients with Mixed Hyperlipidemia and Vulnerable Atherosclerotic Plaque

**DOI:** 10.3390/ph15070802

**Published:** 2022-06-27

**Authors:** Marcin Basiak, Michal Kosowski, Marcin Hachula, Boguslaw Okopien

**Affiliations:** Department of Internal Medicine and Clinical Pharmacology, Medical University of Silesia, Medyków 18, 40-752 Katowice, Poland; mkosowski@sum.edu.pl (M.K.); marcin.hachula@gmail.com (M.H.); bokopien@sum.edu.pl (B.O.)

**Keywords:** PCSK-9 inhibitors, atherosclerotic plaque, hyperlipidemia, interleukin-6, interleukin-18, tumor necrosis factor alfa, metalloproteinase 2

## Abstract

Atherosclerosis is a disorder in which, in addition to high cholesterol levels, several plasma factors play a significant role in its development. Among these cytokines and molecules are interleukin 6 (IL-6), interleukin 18 (IL-18), tumor necrosis factor α (TNF-α), metalloproteinase 2 (MMP-2), and metalloproteinase 9 (MMP-9), all of which may contribute to the stabilization of atherosclerotic plaque. The purpose of this study was to determine the effect of advanced lipid-lowering therapy on the levels of these determinants by utilizing proprotein convertase subtilisin/kexin type 9 (PCSK-9) inhibitors in patients with verified high-risk atherosclerotic plaque. Methods: The study involved patients with dyslipidemia who had the presence of unstable atherosclerotic plaque verified by ultrasonography and who were eligible to begin alirocumab treatment. The levels of IL-6, IL, 18, TNF-α, and MMPs were determined in this group before and after three months of therapy. After treatment, a statistically significant decrease in concentrations of Il-18, Il-6, TNF-α (*p* < 0.001) and MMP-2 (*p* < 0.05) was observed. Additionally, we observed that the concentrations of these markers were significantly higher in the group of patients prior to initiating therapy than in the control group. Our study’s results suggest that PCSK-9 inhibitor therapy significantly reduces the concentration of factors influencing the stability of atherosclerotic plaque, which may explain their essential importance in reducing cardiovascular risk in patients receiving this treatment.

## 1. Introduction

Atherosclerosis is one of the most significant and prevalent causes of peripheral vascular disease, accounting for over 1 million fatalities in European nations in 2016, according to EUROSTAT statistics [1]. It is thought to occur as a persistent inflammatory vascular wall response triggered by endothelial damage. Atherosclerotic plaque development is induced by a chronic inflammatory process that involves both endothelial dysfunction and elevated cholesterol levels in the blood. Foam cells gather in the subepithelial region of the endothelium injured by the inflammatory process, forming an atherosclerotic plaque that, as it grows in size, may completely occlude the artery implicated in the atherosclerotic process [2].

Additionally, it promotes the nuclear translocation of transcription factors encoding pro-inflammatory cytokine genes and inhibits the production of anti-inflammatory cytokines in macrophages [3,4]. Atherosclerotic lesions are associated with a cascade of inflammatory marker secretions and an increase in chemotactic activity. The presence of a substantial number of inflammatory cells, mostly monocytes/macrophages and T lymphocytes, within the atherosclerotic plaque demonstrates that inflammatory processes play a critical role in atherogenesis [5]. Plaques classified as high risk or vulnerable have a higher risk of rupture, uncontrolled embolization, and vascular events. Changes in the histological structure of that plaque reveal a big lipid core and a thin fibrin-coated surface, as well as ulceration, the presence of wall clots or effusion inside, and a high level of macrophage and other inflammatory cell infiltration [6,7]. The significance of biomarkers in detecting sensitive plaque is not clearly known and represents a large topic of research interest, both in terms of cardiovascular disease prevention and response evaluation to anti-atherosclerosis intervention [8,9]. Interleukin 6 (IL-6), interleukin 18 (IL-18), tumor necrosis factor α (TNF-α), metalloproteinase 2 (MMP-2), and metalloproteinase 9 (MMP-9) are examples of these assays [10,11,12,13].

IL-6 has been extensively described in the studies InCHIANTI and MESA as an independent predictor of peripheral artery disease in population screening, regardless of ethnic origin. IL-6 stimulates the expression of adhesive molecules and results in an in-crease in the production and reactivity of acute phase indicators such as C-reactive protein and TNF-α [14,15]. IL-18 is crucial for atherosclerotic plaque development and stability [16]. Exogenous IL-18 administration to mice accelerated the formation of atherosclerotic plaques in experimental studies [17]. TNF-α is present at all stages of atherosclerosis development. It increases adhesion molecule expression, selectin production, and metalloproteinase production in the vascular endothelium [5]. It stimulates the production of tissue factor, a strong thrombogenic protein, locally, within the atherosclerotic plaque [18].

Metalloproteinases (MMPs) are a kind of endopeptidase that cause damage to the extracellular matrix (ECM). They also have a role in the remodeling of arteries [19]. Some of them, particularly MMP-9 and MMP-2, are implicated in the atherogenesis process. As a result, higher concentrations of these MMPs have been seen in the endothelium of arteries during atherogenesis [20]. A high level of MMP-9 has also been associated with an increased risk of plaque rupture in several studies [21].

All of the cytokines mentioned above play a significant role in the formation and destabilization of atherosclerotic plaques and thus can serve as an important biomarker of antiatherosclerosis treatment response, especially given that statin use has been shown to result in a significant decrease in IL-6, TNF-α and MMPs concentrations [22,23].

Inhibitors of proprotein convertase subtilisin/kexin type 9 (PCSK9) are a novel family of cholesterol-lowering medications. PCSK9 has the effect of decreasing the number of low-density lipoprotein cholesterol receptors (LDL-R). PCSK9 inhibitors act by decreasing circulating PCSK9, elevating LDL-R levels, which promotes LDL uptake by the liver and results in a decrease in serum LDL cholesterol levels. Alirocumab and evolocumab have been approved by the Food and Drug Administration for use in clinical practice [24,25]. They are used as monotherapy or in combination with statins or ezetimibe to intensify cholesterol-lowering treatment, according to the American Heart Association and European Society of Cardiology recommendations [26]. Recently, it was shown that the lipid-lowering impact of PCSK9 inhibitors is not the only consequence. The anti-atherosclerotic, atherosclerotic plaque stabilization, anti-aggregation, anticoagulant, and anti-inflammatory effects were described [4,27].

The mechanism by which PCSK9 inhibitors stabilize atherosclerotic plaque is extremely complicated, including many signaling pathways related to pro-inflammatory cytokines such as TNF-α, interleukin 1 (IL-1), and IL-6 [28]. Although the mechanism by which PCSK9 inhibitors alter MMPs expression is not fully known, it is likely related to PCSK9’s direct influence on the synthesis of ECM proteins by endothelial and vascular smooth muscle cells’ endoplasmic reticulum [28,29]. Additionally, the effect of new lipid-lowering therapy with PCSK9 inhibitors on IL-18 and MMPs levels is unknown. The purpose of this study was to determine if PCSK9 inhibitors have any impact on the concentration of markers of atherosclerotic plaque vulnerability in hyperlipidemic patients.

## 2. Results

In the study group, no significant differences in terms of demographic data (age, gender, smoking and weight) were observed (Table 1). After reviewing patient records, it was discovered that patients in both groups had not received any medications in the three months preceding enrollment.

In the control group, we observed statistically significantly lower concentrations of total cholesterol (TC), LDL, non-high-density lipoprotein cholesterol (non-HDL) and triglycerides (TG) (*p* < 0.001), and a higher concentration of HDL (*p* < 0.05) than in the study group before treatment. Moreover, after treatment, a statistically significant decrease in TC, LDL, non-HDL and TG concentrations and an increase in HDL concentration could be observed (*p* < 0.001) (Table 2 and Table 3).

In the study group, according to the adopted classification, all patients met the criteria for qualifying atherosclerotic plaques into the category of unstable plaques. An ultrasound image of the surface of the atherosclerotic plaque, i.e., the presence of irregularities or ulcerations, helps in determining its state of stability. It therefore completes the description of the echogenicity on the Gray–Weale–Nicolaides scale. The following were observed in the group of 21 enrolled patients: 14 patients (predominately echolucent), including 10 with irregularities and four with ulcerations; the part of the plaque affected by calcification did not exceed 25% of the plaque volume (assessed in a volumetric test), or 20–25% of the plaque size (2D assessment); seven patients (predominately echogenic), including five with irregularities and two with ulcerations; the composition of the plaque with the presence of numerous calcifications.

In these subgroups of plaques, using the imaging option flow using Doppler or non-Doppler methods, the classification of irregularities is facilitated on the surface of the plates, with the possibility of visualizing any irregularities or ulcerations.

We also compared the concentrations of the mediators, which we tested between the control group and the study group before treatment. We observed that in the control group, concentrations of IL-18 (*p* < 0.01), IL-6 (*p* < 0.01), MMP-2 (*p* < 0.001) and MMP-9 (*p* = 0.001) were statistically significantly lower than in the study group before treatment. There were no statistically significant differences in TNF-α concentrations between these two groups.

**Figure 1:**
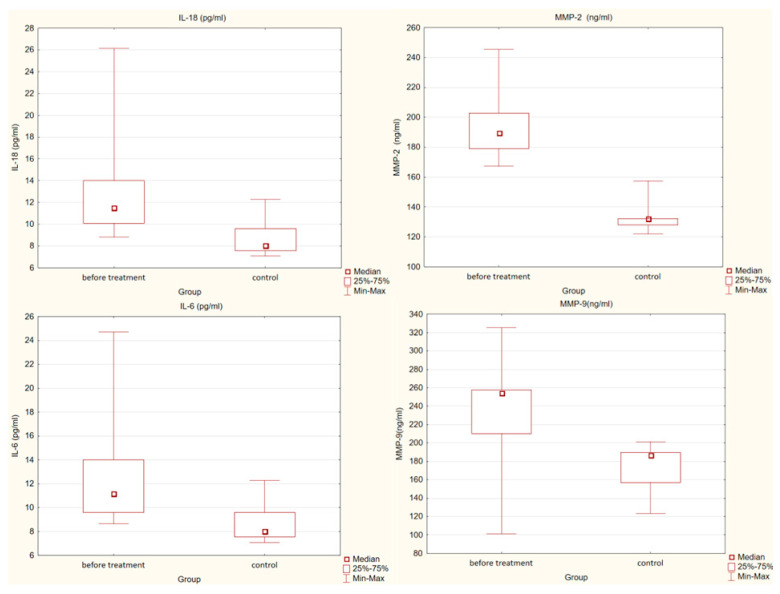
Differences in concentrations of interleukin 18 (IL-18), interleukin 6 (IL-6) (*p* < 0.01), metalloproteinase 2 (MMP-2) (*p* < 0.001) and metalloproteinase 9 (MMP-9) (*p* = 0.001) in the study and control groups.

Detailed results are presented in Figure 1.

We also assessed the effect of treatment with PCSK9 inhibitors on the concentrations of the individual mediators mentioned above. After treatment, a statistically significant decrease in concentrations of IL-18, IL-6, TNF-α (*p* < 0.001) and MMP-2 (*p* < 0.05) was observed. There were no statistically significant differences in MMP-9 concentrations before and after treatment.

**Figure 2:**
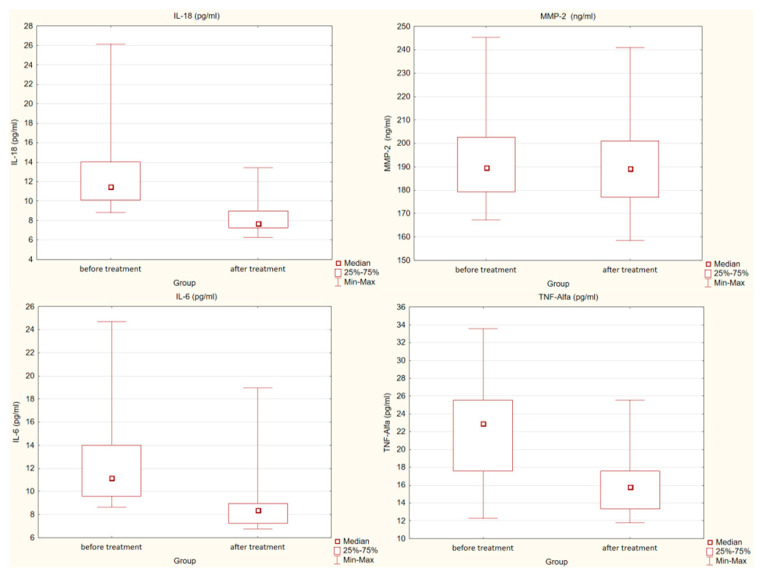
Differences in concentration of interleukin 18 (IL-18), interleukin 6 (IL-6), tumor necrosis factor α (TNF-α) (*p* < 0.001) and metalloproteinase 2 (MMP-2) (*p* < 0.05) in the study group before and after treatment.

Figure 2 present detailed results.

## 3. Discussion

Atherosclerosis and its complications in the form of major cardiovascular incidents are an increasingly frequent cause of death [30]. It is the reason that the investigation for such biochemical parameters was started, which will enable even better estimation of the risk of a cardiovascular event in patients compared to the lipid profile determinations used thus far. The researchers’ attention is particularly focused on cytokines and proteins that may be responsible for the stabilization of atherosclerotic plaque and thus influence this risk.

Numerous cytokines, including those researched by our department, are implicated in the etiology of atherosclerosis. Additionally, they are involved in the synthesis of MMPs, which are implicated in the processes that damage atherosclerotic plaques by degrading ECM [31,32].

Due to the complicated pathophysiology of the atherosclerotic process, investigations were conducted at the beginning of the century to establish the influence of previously extensively used lipid-lowering treatment with statins on additional parameters impacting cardiovascular disease (CVD) risk. It has been demonstrated that this medication affects not only cholesterol concentrations, but also pro-inflammatory factor concentrations, resulting in a higher decrease in CVD risk than only reducing LDL concentrations [33,34]. Furthermore, additional research in people and animal models has found that statin treatment reduces proinflammatory cytokines, MMP-2, and MMP-9 levels considerably [35,36,37,38]. These treatments reduce the risk of cardiovascular disease by stabilizing the atherosclerotic plaque. These findings prompted researchers to investigate whether modern lipid-lowering therapies, such as those involving PCSK9 inhibitors, have similar pleiotropic effects, particularly since studies such as the CANTOS and FOURIER studies have shown that CVD risk during treatment with these drugs decreases irrespectively to the reduction in LDL concentration [39]. PCSK9 inhibitors have been shown to improve the stability and structure of atherosclerotic plaques, making them less vulnerable [40,41]. Additionally, they enhance platelet function by decreasing their thrombogenic capacity [42,43]. According to studies using intravascular ultrasonography, the concentration of PCSK9 has an effect on the size of the necrotic core within the atherosclerotic plaque, regardless of the LDL-C concentration [44]. In 2020, more extensive research revealed that while PCSK9 medications do not impact the overall size of the atherosclerotic plaque, they considerably improve its stabilization by lowering the lipid core burden index [45].

Inflammatory processes are critical in atherosclerosis pathophysiology [46]. Their escalation is connected with atherogenesis. PCSK9 has been shown to induce the expression of pro-inflammatory cytokines such as TNF-α and IL-6 [3]. Similar results were obtained in our study. Additionally, it promotes the nuclear translocation of transcription factors encoding pro-inflammatory cytokine genes and inhibits the production of anti-inflammatory cytokines in macrophages [3,4].

A critical mechanism of action for new lipid-lowering therapies with pleiotropic effects that inhibit monocyte diapedesis into atherosclerotic plaques is elevated anti-inflammatory interleukin 10 (IL-10) concentration. Increases in its concentration result in a decrease in TNF-α expression, which is responsible for monocyte influx into the atherosclerotic plaque [40,47].

To the best of our knowledge, no studies have been conducted to date that reveal the effect of PCSK9 inhibitors on IL-18 and MMPs concentrations. Only one animal research, completed by Elsweidy M.M et al., demonstrated that polyconasol, a medication that lowers PCSK9 concentration, can lower osteopontin and MMPs concentrations in atherosclerotic plaques [48]. IL-18 as the major pro-inflammatory cytokine may reflect the situation of the inflammatory response. Previous reports have shown that IL-18 may promote the progression of atherosclerosis, destabilize atherosclerotic plaque and accelerate plaque formation [49]. Youssef et al. showed that elevated plasma levels of IL-18 may be an important independent predictor of adverse clinical events within 30 days in ST-elevation myocardial infarction STEMI patients [50]. Serum IL-18 played a major role in the establishment and development of atherosclerotic plaques in animal models of atherosclerosis and was associated with the plaque’s stability and severity [51]. This finding is in accordance with our findings, which show a decrease in the concentrations of TNF-α, IL-6, IL-18 and MMP-2 following the administration of PCSK9 inhibitors. Furthermore, plasma-stabilizing factors for atherosclerotic plaque were identified in one of Otake H.’s investigations, which included patients treated with alirocumab. The authors, however, do not discuss the impact of alirocumab on these parameters in this investigation, instead focusing on the favorable effect of this medication on plaque vulnerability as measured by optical coherence tomography [52].

### The Current Study’s Limitations

The experiment was short and did not analyze clinical outcomes, and the study population was small, while exceeding the needed sample size. However, this was due to the fact that the number of patients treated with PCSK9 inhibitors in our country is rather low, and we chose specific and difficult criteria to meet. The lack of a placebo group was the second issue. This was due to the fact that delaying PCSK9 inhibitor treatment in this group of patients would be unethical. The short follow-up period of only three months may possibly be a significant limitation. Our experiment focused on interleukins and cytokines as new markers, and we did not include analysis of the classic inflammatory markers such as hsCRP, fibrinogen, and white blood cell count. As a result, we believe that additional research using a bigger sample size is necessary to definitively establish the effect of PCSK9 inhibitor therapy on proinflammatory cytokines and novel atherosclerosis markers.

## 4. Materials and Methods

The study population was recruited from individuals aged 18–75 years who were tested in our department for the existence of asymptomatic atherosclerosis using sonographic assessment of common carotid intima-media thickness.

The following criteria were used to assess if a subject was eligible for the study: mixed hyperlipidemia (previously Frederickson hyperlipidemia type 2B)—plasma TC > 200 mg/dL, LDL > 135 mg/dL, and TG > 150 mg/dL; for women at least 24 months since the last menstruation, hysterectomy or ovarectomy, or using mechanical contraception.

The exclusion criteria were as follows: other types of primary dyslipidemias; secondary dyslipidemia in the course of autoimmune disorders, thyroid diseases, chronic pancreatitis, nephrotic syndrome, liver and biliary tract diseases, obesity (body mass index >30 kg/m^2^) or alcoholism; any acute and chronic inflammatory processes; symptomatic congestive heart failure; unstable coronary artery disease, myocardial infarction or stroke within 6 months preceding the study; arterial hypertension; impaired renal or hepatic function; malabsorption syndromes; treatment with other hypolipidemic drugs within 3 months before the study; concomitant treatment with other drugs known either to affect plasma lipid levels; concomitant treatment with drugs that may affect inflammatory processes in the vascular wall within 3 months before the enrollment; ongoing hormonal replacement therapy or oral contraception.

### 4.1. Specific Inclusion Criteria for Atherosclerotic Plaque with a High Risk of Rupture

Ultrasonography is a technique for evaluating atherosclerotic carotid disease; it is used clinically to determine the presence of plaque, the degree of carotid stenosis as determined by blood-flow velocity profiles, and the carotid intima-media thickness. The examination of the carotid arteries and assessment of complex intima media thickness (C-IMT) in the extracranial segment was performed using Hitachi Aloka Arietta S60 ultrasound with a linear probe at a frequency of 7.5–10 MHz. As previously described, unstable plaques were related with fibrofatty and hemorrhagic material and had an echolucent appearance. [6]. Additionally, high-risk plaques may have an irregular surface or ulcerations, which the Color-Doppler approach discovered [7].

Of the 128 patients screened, 21 met all of the study’s rigorous inclusion criteria and were eligible for enrollment.

Each patient provided written informed consent in accordance with the Helsinki Declaration. The Medical University of Silesia’s Bioethical Committee accepted the study protocol. Due to the established value of lipid-lowering therapy, the patients were thereafter treated with high-effective lipid-lowering therapy. Our control group consisted of 12 healthy volunteers who were age, sex, and weight matched.

Prior to initiating therapy, medical history and clinical examination was performed, and venous blood was obtained to evaluate safety laboratory values. Total and differential blood cell counts, blood sedimentation rate, creatine kinase, alanine and aspartate aminotransferases, gamma-glutamyltransferase, alkaline phosphatase, electrolytes, bilirubin, creatinine, total proteins, urine examination, glycated hemoglobin, and 12-lead electrocardiogram were among the tests performed.

Venous blood samples were taken at 8 a.m. prior to the treatment following a 12 h fast. All tests were conducted by a person who was unaware of the subject’s identity or any clinical details. Plasma lipids and glucose levels were determined using standard laboratory procedures, while LDL cholesterol levels were determined directly. Plasma levels of interleukins, cytokines and metalloproteinases were determined using commercially available enzyme immunoassay kits Cloud-Clone Corp., Houston, TX, USA (Human IL-18—SEA064Hu 96, L170622820; MMP-2—SEA100Hu 96, L190730467); and Diaclone, France (Human IL-6 ELISA Kit—950.030.096; Human TNF alfa Elisa Kit—950.090.096); and BioVendor R&D Czech Republic (Human MMP-9—RD191439100CS) respectively. All laboratory tests were conducted on the control group as well. Each experiment was performed on a single sample aliquot to prevent the freeze–thaw effect.

### 4.2. Statistical Analysis

The collected data were processed via the Statistica TIBCO Software Inc. Palo Alto, Santa Clara, CA, USA (2017) version 13.3 program, licensed by the Medical University of Silesia in Katowice. The normality of distributions was assessed by the Shapiro–Wilk test. To compare quantitative variables with normal distribution (concentration of TC, LDL, HDL and non-HDL), the *t* test for dependent means was used. For dependent variables with abnormal distribution (concentration of TG, interleukins, metalloproteinases and TNF-α), we used the Wilcoxon test. To compare independent variables with normal distribution (plasma lipid levels) the *t* test for independent means was used, and for variables with abnormal distribution (concentration of metalloproteinases, interleukins and TNF-α) we used U Mann–Whitney test. We assumed a *p* value of less than 0.05 as statistically significant.

## 5. Conclusions

PCSK9 inhibitor therapy decreases the concentrations of MMPs, IL-6, IL-18, TNF-α, all of which may contribute to the stability of atherosclerotic plaque. Additional research is required to definitively assess the effect of the novel lipid-lowering therapy on the levels of atherosclerotic biomarkers and plaque rupture risk.

## Figures and Tables

**Table 1:** Baseline characteristics of patients (values are mean ± SD unless indicated otherwise).

	Control Group	Study Group before Treatment
Number of patients	12	21
Age, years	45 ± 5	47 ± 5
Women, %	37	35
BMI	27.4 ± 2.7	28.1 ± 2.2
WHO guidelines on physical activity, %	84	81
Smokers, %	26	24
Alcohol abuse	No	No
Systolic blood pressure, mmHg	132 ± 5	133 ± 6
Diastolic blood pressure, mmHg	84 ± 3	84 ± 4
Fasting glucose, mg/dL	91 ± 5	92 ± 5
White blood cell count, ×10^9^/L	5.3 ± 1.2	8.2 ± 1.6
High-sensitivity C-reactive protein, mg/L	1.84 ± 0.98	2.87 ± 1.12

**Table 2:** Concentrations of plasma lipids in study group before treatment versus control group. TC—total cholesterol; LDL—low-density lipoprotein cholesterol; HDL—high-density lipoprotein cholesterol; non-HDL—non-high-density lipoprotein cholesterol; TG—triglycerides; Q1—first quartile; Q3—third quartile.

	Control			Study Group before Treatment			
	**Median**	**Q1**	**Q3**	**Median**	**Q1**	**Q3**	
**TG (mg/dL)**	121.1	99.8	142.2	178.4	168	198.8	<0.001
**TC (mg/dL)**	168.2	151.1	174.6	246.2	224.7	278.2	<0.001
**LDL (mg/dL)**	94.7	89.8	105.6	167.6	155.5	201.4	<0.001
**HDL (mg/dL)**	47.1	41.1	48.3	41	34.2	43.1	<0.05
**non-HDL (mg/dL)**	121.1	105.4	133.2	203.2	188.3	246.1	<0.001

**Table 3:** Effect of proprotein convertase subtilisin/kexin type 9 (PCSK-9) inhibitors on plasma lipids. TC—total cholesterol; LDL—low-density lipoprotein cholesterol; HDL—high-density lipoprotein cholesterol; non-HDL—non-high-density lipoprotein cholesterol; TG—triglycerides; SD—standard deviation; Q1—first quartile; Q3—third quartile.

	Study Group before Treatment			Study Group after Treatment			
	Mean	SD		Mean	SD		*p*
**TC (mg/dL)**	250.6	35.4		173.8	31.6		<0.001
**LDL (mg/dL)**	172.7	30		94.4	29.1		<0.001
**HDL (mg/dL)**	40.3	10.1		49.5	10.3		<0.001
**non-HDL (mg/dL)**	210.3	34.2		124.3	34.9		<0.001
	**Median**	**Q1**	**Q3**	**Median**	**Q1**	**Q3**	
**TG (mg/dL)**	178.4	168	198.8	142.8	123.8	157.1	<0.001

## Data Availability

Data is contained within the article.

## References

[1] Causes and Occurrence of Deaths in the EU. https://ec.europa.eu/eurostat/web/products-eurostat-news/product/-/asset_publisher/VWJkHuaYvLIN/content/DDN-20190716-1/pop_up.

[2] Ross R. (1998). Atherosclerosis—An inflammatory disease. N. Engl. J. Med..

[3] Ricci C., Ruscica M., Camera M., Rossetti L., Macchi C., Colciago A., Zanotti I., Lupo M.G., Adorni M.P., Cicero A.F. (2018). PCSK9 induces a pro-inflammatory response in macrophages. Sci. Rep..

[4] Tang Z.H., Peng J., Ren Z., Yang J., Li T.T., Li T.H., Wang Z., Wei D.H., Liu L.S., Zheng X.L. (2017). New role of PCSK9 in atherosclerotic inflammation promotion involving the TLR4/NF-κB pathway. Atherosclerosis.

[5] Young J.L., Libby P., Schonbeck U. (2002). Cytokines in the pathogenesis of atherosclerosis. Thromb. Haemost..

[6] Mathiesen E.B., Bonaa K.H., Joakimsen O. (2001). Echolucent plaques are associated with high risk of ischemic cerebrovascular events in carotid stenosis: The Tromso Study. Circulation.

[7] Prabhakaran S., Rundek T., Ramas R., Elkind M.S., Paik M.C., Boden-Albala B., Sacco R.L. (2006). Carotid plaque surface irregularity predicts ischemic stroke: The Northern Manhattan Study. Stroke.

[8] Thim T., Hagensen M.K., Bentzon J.F., Falk E. (2008). From vulnerable plaque to atherothrombosis. J. Intern. Med..

[9] Naghavi M., Libby P., Falk E., Casscells S.W., Litovsky S., Rumberger J., Badimon J.J., Stefanadis C., Moreno P., Pasterkamp G. (2003). From vulnerable plaque to vulnerable patient: A call for new definitions and risk assessment strategies: Part I. Circulation.

[10] Lyngbakken M.N., Myhre P.L., Røsjø H., Omland T. (2019). Novel biomarkers of cardiovascular disease: Applications in clinical practice. Crit. Rev. Clin. Lab. Sci..

[11] Bahrami A., Sathyapalan T., Sahebkar A. (2021). The Role of Interleukin-18 in the Development and Progression of Atherosclerosis. Curr. Med. Chem..

[12] Santana I.V., Tanus-Santos J.E. (2018). Serum or Plasma Matrix Metalloproteinase (MMP)-9 Levels and Cardiovascular Diseases. J. Cardiovasc. Transl. Res..

[13] Ain Q.U., Sarfraz M., Prasesti G.K., Dewi T.I., Kurniati N.F. (2021). Confounders in Identification and Analysis of Inflammatory Biomarkers in Cardiovascular Diseases. Biomolecules.

[14] McDermott M.M., Guralnik J.M., Corsi A., Albay M., Macchi C., Bandinelli S., Ferrucci L. (2005). Patterns of inflammation associated with peripheral arterial disease: The InCHIANTI study. Am. Heart J..

[15] Allison M.A., Criqui M.H., McClelland R.L., Scott J.M., McDermott M.M., Liu K., Folsom A.R., Bertoni A.G., Sharrett A.R., Homma S. (2006). The effect of novel cardiovascular risk factors on the ethnic-specific odds for peripheral arterial disease in the Multi-Ethnic Study of Atherosclerosis (MESA). J. Am. Coll. Cardiol..

[16] Mallat Z., Corbaz A., Scoazec A., Besnard S., Lesèche G., Chvatchko Y., Tedgui A. (2001). Expression of interleukin-18 in human atherosclerotic plaques and relation to plaque instability. Circulation.

[17] Elhage R., Jawien J., Rudling M., Ljunggren H.-G., Takeda K., Akira S., Bayard F., Hansson G.K. (2003). Reduced atherosclerosis in interleukin-18 deficient apolipoprotein E-knockout mice. Cardiovasc. Res..

[18] Taubman M.B., Fallon J.T., Schecter A.D., Giesen P., Mendlowitz M., Fyfe B.S., Marmur J.D., Nemerson Y. (1997). Tissue factor in the pathogenesis of atherosclerosis. Thromb. Haemost..

[19] Lessner S.M., Galis Z.S. (2004). Matrix metalloproteinases and vascular endothelium-mononuclear cell close encounters. Trends Cardiovasc. Med..

[20] Choudhary S., Higgins C.L., Chen I.Y., Reardon M., Lawrie G., Vick G.W., Karmonik C., Via D.P., Morrisett J.D. (2006). Quantitation and localization of matrix metalloproteinases and their inhibitors in human carotid endarterectomy tissues. Arterioscler. Thromb. Vasc. Biol..

[21] Zouridakis E., Avanzas P., Arroyo-Espliguero R., Fredericks S., Kaski J.C. (2004). Markers of inflammation and rapid coronary artery disease progression in patients with stable angina pectoris. Circulation.

[22] Kadoglou N., Kottas G., Lampropoulos S., Vitta I., Liapis C. (2014). Serum Levels of Fetuin-A, Osteoprotegerin and Osteopontin in Patients with Coronary Artery Disease: Effects of Statin (HMGCoA-Reductase Inhibitor) Therapy. Clin. Drug Investig..

[23] Fujimoto S., Hartung D., Ohshima S., Edwards D.S., Zhou J., Yalamanchili P., Azure M., Fujimoto A., Isobe S., Matsumoto Y. (2008). Molecular imaging of matrix metalloproteinase in atherosclerotic lesions: Resolution with dietary modification and statin therapy. J. Am. Coll. Cardiol..

[24] Ingueneau C., Hollstein T., Grenkowitz T., Ruidavets J.-B., Kassner U., Duparc T., Combes G., Perret B., Genoux A., Schumann F. (2020). Treatment with PCSK9 inhibitors induces a more anti-atherogenic HDL lipid profile in patients at high cardiovascular risk. Vascul. Pharmacol..

[25] Saborowski M., Dölle M., Manns M.P., Leitolf H., Zender S. (2018). Lipid-lowering therapy with pcsk9-inhibitors in the management of cardiovascular high-risk patients: Effectiveness, therapy adherence and safety in a real world cohort. Cardiol. J..

[26] Stein E.A., Gipe D., Bergeron J., Gaudet D., Weiss R., Dufour R., Wu R., Pordy R. (2012). Effect of a monoclonal antibody to PCSK9, REGN727/SAR236553, to reduce low-density lipoprotein cholesterol in patients with heterozygous familial hypercholesterolaemia on stable statin dose with or without ezetimibe therapy: A phase 2 randomised controlle. Lancet.

[27] Basiak M., Kosowski M., Cyrnek M., Bułdak Ł., Maligłówka M., Machnik G., Okopień B. (2021). Pleiotropic Effects of PCSK-9 Inhibitors. Int. J. Mol. Sci..

[28] Karagiannis A.D., Liu M., Toth P.P., Zhao S., Agrawal D.K., Libby P., Chatzizisis Y.S. (2018). Pleiotropic Anti-atherosclerotic Effects of PCSK9 InhibitorsFrom Molecular Biology to Clinical Translation. Curr. Atheroscler. Rep..

[29] Bennett M.R., Sinha S., Owens G.K. (2016). Vascular Smooth Muscle Cells in Atherosclerosis. Circ. Res..

[30] Virani S.S., Alonso A., Aparicio H.J., Benjamin E.J., Bittencourt M.S., Callaway C.W., Carson A.P., Chamberlain A.M., Cheng S., Delling F.N. (2021). American Heart Association Council on Epidemiology and Prevention Statistics Committee and Stroke Statistics Subcommittee. Heart Disease and Stroke Statistics-2021 Update: A Report from the American Heart Association. Circulation.

[31] Siasos G., Tousoulis D., Kioufis S., Oikonomou E., Siasou Z., Limperi M., Papavassiliou A.G., Stefanadis C. (2012). Inflammatory mechanisms in atherosclerosis: The impact of matrix metalloproteinases. Curr. Top. Med. Chem..

[32] Amin M., Pushpakumar S., Muradashvili N., Kundu S., Tyagi S.C., Sen U. (2016). Regulation and involvement of matrix metalloproteinases in vascular diseases. Front. Biosci. (Landmark Ed.).

[33] Albert M.A., Danielson E., Rifai N., Ridker P.M., PRINCE Investigators (2001). Effect of statin therapy on C-reactive protein levels: The pravastatin inflammation/CRP evaluation (PRINCE): A randomized trial and cohort study. JAMA.

[34] Goettsch C., Hutcheson J.D., Hagita S., Rogers M., Creager M.D., Pham T., Choi J., Mlynarchik A.K., Pieper B., Kjolby M. (2016). A single injection of gain-of-function mutant PCSK9 adeno-associated virus vector induces cardiovascular calcification in mice with no genetic modification. Atherosclerosis.

[35] Hwang H.S., Kim J.S., Kim Y.G., Lee S.Y., Ahn S.Y., Lee H.J., Lee D.Y., Lee S.H., Moon J.Y., Jeong K.H. (2020). Circulating PCSK9 Level and Risk of Cardiovascular Events and Death in Hemodialysis Patients. J. Clin. Med..

[36] Lupo M.G., Bressan A., Donato M., Canzano P., Camera M., Poggio P., Greco M.F., Garofalo M., De Martin S., Panighel G. (2022). PCSK9 promotes arterial medial calcification. Atherosclerosis.

[37] Golia E., Limongelli G., Natale F., Fimiani F., Maddaloni V., Pariggiano I., Bianchi R., Crisci M., D’Acierno L., Giordano R. (2014). Inflammation and cardiovascular disease: From pathogenesis to therapeutic target. Curr. Atheroscler. Rep..

[38] Guo H., Shi Y., Liu L., Sun A., Xu F., Chi J. (2009). Rosuvastatin inhibits MMP-2 expression and limits the progression of atherosclerosis in LDLR-deficient mice. Arch. Med. Res..

[39] Ridker P.M. (2018). Mortality differences associated with treatment responses in CANTOS and FOURIER: Insight and implications. Circulation.

[40] Kühnast S., Van Der Hoorn J.W., Pieterman E.J., van den Hoek A.M., Sasiela W.J., Gusarova V., Peyman A., Schäfer H.L., Schwahn U., Jukema J.W. (2014). Alirocumab inhibits atherosclerosis, improves the plaque morphology, and enhances the effects of a statin. J. Lipid Res..

[41] Yano H., Horinaka S., Ishimitsu T. (2020). Effect of evolocumab therapy on coronary fibrous cap thickness assessed by optical coherence tomography in patients with acute coronary syndrome. J. Cardiol..

[42] Barale C., Bonomo K., Frascaroli C., Morotti A., Guerrasio A., Cavalot F., Russo I. (2020). Platelet function and activation markers in primary hypercholesterolemia treated with anti-PCSK9 monoclonal antibody: A 12-month follow-up. Nutr. Metab. Cardiovasc. Dis..

[43] Koskinas K.C., Windecker S., Buhayer A., Gencer B., Pedrazzini G., Mueller C., Cook S., Muller O., Matter C.M., Räber L. (2018). Design of the randomized, placebo-controlled evolocumab for early reduction of LDL-cholesterol levels in patients with acute coronary syndromes (EVOPACS) trial. Clin. Cardiol..

[44] Cheng J.M., Oemrawsingh R.M., Garcia-Garcia H.M., Boersma E., van Geuns R.J., Serruys P.W., Kardys I., Akkerhuis K.M. (2016). PCSK9 in relation to coronary plaque inflammation: Results of the ATHEROREMOIVUS study. Atherosclerosis.

[45] Omori H., Ota H., Hara M., Kawase Y., Tanigaki T., Hirata T., Sobue Y., Okubo M., Kamiya H., Matsuo H. (2020). Effect of PCSK-9 Inhibitors on Lipid-Rich Vulnerable Coronary Plaque Assessed by Near-Infrared Spectroscopy. JACC Cardiovasc. Imaging.

[46] Geovanini G.R., Libby P. (2018). Atherosclerosis and inflammation: Overview and updates. Clin. Sci..

[47] Schwartz G.G., Steg P.G., Szarek M., Bittner V.A., Diaz R., Goodman S.G., Kim Y.U., Jukema J.W., Pordy R., Roe M.T. (2020). Peripheral Artery Disease and Venous Thromboembolic Events after Acute Coronary Syndrome: Role of Lipoprotein(a) and Modification by Alirocumab: Prespecified Analysis of the ODYSSEY OUTCOMES Randomized Clinical Trial. Circulation.

[48] Elseweidy M.M., Mohamed H.E., Elrashidy R.A., Atteia H.H., Elnagar G.M. (2018). Inhibition of Aortic Calcification by Policosanol in Dyslipidemic Rabbits Is Enhanced by Pentoxifylline: Potential Role of PCSK9. J. Cardiovasc. Pharmacol. Ther..

[49] de Nooijer R., von der Thüsen J., Verkleij C., Kuiper J., Jukema J., van der Wall E., van Berkel T., Biessen E. (2004). Overexpression of IL-18 decreases intimal collagen content and promotes a vulnerable plaque phenotype in apolipoprotein-E-deficient mice. Arterioscler. Thromb. Vasc. Biol..

[50] Youssef A.A., Chang L.-T., Wu C.-J., Cheng C.-I., Yang C.-H., Sheu J.-J., Chai H.-T., Chua S., Yeh K.-H., Yip H.-K. (2007). Level and value of interleukin-18 in patients with acute myocardial infarction undergoing primary coronary angioplasty. Circ. J. Off. J. Jpn. Circ. Soc..

[51] Tang X. (2019). Analysis of interleukin-17 and interleukin-18 levels in animal models of atherosclerosis. Exp. Ther. Med..

[52] Otake H., Sugizaki Y., Toba T., Nagano Y., Tsukiyama Y., Yanaka K.I., Yamamoto H., Nagasawa A., Onishi H., Takeshige R. (2019). Efficacy of alirocumab for reducing plaque vulnerability: Study protocol for ALTAIR, a randomized controlled trial in Japanese patients with coronary artery disease receiving rosuvastatin. J. Cardiol..

